# Bacteriophages with Potential for Inactivation of Fish Pathogenic Bacteria: Survival, Host Specificity and Effect on Bacterial Community Structure

**DOI:** 10.3390/md9112236

**Published:** 2011-11-07

**Authors:** Carla Pereira, Yolanda J. Silva, Ana L. Santos, Ângela Cunha, Newton C. M. Gomes, Adelaide Almeida

**Affiliations:** CESAM and Department of Biology, Campus of Santiago, University of Aveiro, 3810-193 Aveiro, Portugal; E-Mails: csgp@ua.pt (C.P.); yolanda@ua.pt (Y.J.S.); alsantos@ua.pt (A.L.S.); acunha@ua.pt (Â.C.); gomesncm@ua.pt (N.C.M.G.)

**Keywords:** phage therapy, bacteriophages, fish pathogenic bacteria, aquaculture, bacterial community structure

## Abstract

Phage therapy may represent a viable alternative to antibiotics to inactivate fish pathogenic bacteria. Its use, however, requires the awareness of novel kinetics phenomena not applied to conventional drug treatments. The main objective of this work was to isolate bacteriophages with potential to inactivate fish pathogenic bacteria, without major effects on the structure of natural bacterial communities of aquaculture waters. The survival was determined in marine water, through quantification by the soft agar overlay technique. The host specificity was evaluated by cross infection. The ecological impact of phage addition on the structure of the bacterial community was evaluated by DGGE of PCR amplified 16S rRNA gene fragments. The survival period varied between 12 and 91 days, with a higher viability for *Aeromonas salmonicida* phages. The phages of *Vibrio parahaemolyticus* and of *A. salmonicida* infected bacteria of different families with a high efficacy of plating. The specific phages of pathogenic bacteria had no detectable impact on the structure of the bacterial community. In conclusion, *V. parahaemolyticus* and *A. salmonicida* phages show good survival time in marine water, have only a moderated impact on the overall bacterial community structure and the desired specificity for host pathogenic bacteria, being potential candidates for therapy of fish infectious diseases in marine aquaculture systems.

## 1. Introduction

Nearly one-third of the world’s seafood supplies come from aquaculture industry, representing the fastest growing agricultural sector. Over the past ten years, aquaculture production has increased on average by 6% per year [1]. The production has increased from 8.7 million tons of fish in 1990 to 31.6 million tons in 2006 [1,2]. Fish farming plants, however, often suffer from heavy financial losses [3–5] due to the development of infections caused by microbial pathogens, including multidrug resistant bacteria that are easily transmitted through water and therefore able to infect a great variety of fish species.

Although pathogenic species have been described in the majority of the existing taxonomic groups, only a relatively small number is responsible for important economic losses in cultured fish worldwide [6]. Vibriosis and photobacteriosis are primarily diseases of marine and estuarine fish, both in natural and commercial production systems throughout the world, occurring only occasionally in freshwater fish. Both diseases can cause significant mortality in fish, reaching values of up to 100% in infected facilities, being currently responsible for most outbreaks in fish farming plants. Vibriosis and photobacteriosis are caused by bacteria from the family Vibrionaceae. Vibriosis is caused by species of *Vibrio*, namely by *Vibrio anguillarum* [7–9]. Others species of *Vibrio*, such as *V. alginolitycus*, *V. anguillarum*, *V. carchariae*, *V. salmonicida*, *V. damsela*, *V. ordalii*, *V. parahemolyticus* and *V. vulnificus*, causes also important infections in several species of fish [10,11]. Photobacteriosis is caused by *Photobacterium damselae* subsp. *piscicida* (formerly *Pasteurella piscicida*) which is a highly pathogenic bacterium that does not seem to have host specificity, infecting a diverse range of fish species [6,12]. Other bacteria as *A. salmonicida*, causative agent of furunculosis, *Rickettsia*-like bacteria, *Cytophaga marina*, *Flavobacterium psychrophilum* and *Pseudomonas plecoglossicida* are also important groups of fish pathogens, affecting a variety of fish species from diverse geographical aquatic environments [13,14].

Although vaccination is the ideal method to prevent infectious diseases [15–18], many different kinds of infectious diseases occur locally in a variety of fish species [19], thus limiting its application. On the other hand, chemotherapy is a rapid and effective method to treat or prevent bacterial infections, but the frequent use of antibiotics has resulted in an increasing frequency of drug-resistant pathogenic bacteria in the aquaculture, agriculture, and medical realms. This problem can be particularly serious since few chemotherapeutic drugs are licensed for fisheries use [19–21].

To reduce the risk of development and spreading of antibiotic resistant bacteria, other more environmentally friendly methods to control fish disease in aquaculture must be developed. In line with this idea, the use of phage therapy in aquaculture seems to be very promising, as bacterial diseases are a major problem in the expanding aquaculture industry [22–24].

Bacteriophages (commonly phages) are bacterial viruses extremely abundant in nature and believed to be important in controlling bacterial populations in natural systems [25], even being multidrug resistant [26–28]. The use of phages to control infections in aquatic environment, such as fish diseases, seems to be particularly promising [19,29,30]. As the host fish organisms live in aqueous media, the therapeutic phages can have continuous and close physiological contact with the pathogens in a natural arrangement. In fact, the potential use of phage therapy to control disease in aquaculture systems has been demonstrated in some studies [19,31–34]. Isolated phages with lytic activity against *Vibrio harveyi* were applied as phage therapy against luminous vibriosis to improve the survival of *Penaeus monodon* larvae [34]. The interaction between *A. salmonicida* and a bacteriophage, to treat furunculosis in brook trout, showed that bacteriophage combination could be successfully used in preventive programs in fish farms [31]. This study showed that more than one phage could infect *A. salmonicida* and that mutants resistant to one phage were sensitive to one or more phages. More than 25% of the mutants seemed to revert to the original strain phenotype after the first plating. All mutants were sensitive to three or more phages [31].

The success of phage therapy to control pathogenic bacteria of fish depends on viral survival and viability in culture water of fish-farming plants. However, although there are some data available on the mechanisms and rates of mortality or loss of infectivity of phages in marine waters, little is known about their time of survival in the marine environment. Solar radiation has been recognized as the most important factor in the loss of phage infectivity in surface coastal water. Suttle and coworkers have examined several processes implicated in the loss of infectivity of viruses in seawater. They used various indigenous marine bacteriophages [35,36], cyanophage [35] and a virus of *Micromonas pusilla*, a cosmopolitan marine phytoplankton [37] and concluded that sunlight was the dominant factor controlling decay of viral infectivity in seawater. De Paepe and Taddei [38] by comparing life history traits of 16 phages infecting the bacterium *Escherichia coli*, showed that their mortality rate is constant with time and negatively correlated to their multiplication rate in the bacterial host. The authors showed that the capsid thickness and the density of the packaged genome account for 82% of the variation in the mortality rate [38].

The success of phage therapy also depends on the effect of phages on the bacterial community, namely when semi-intensive regimes are used. In semi-intensive aquaculture systems, non-pathogenic bacteria have a central role in the functioning and productivity of these ecosystems. Bacteria are the most important biological component involved in the turnover of organic matter in aquatic systems [39,40]. Although the host specificity of many phages is likely to restrict their impact on the diverse members of a microbial assemblage, phage therapy would require adding massive quantities of phages to water. Therefore, a careful evaluation on the impact of any phage on the bacterial community structure should be conducted prior to implementation of phage therapy. Previous studies have demonstrated a huge diversity of both phages and hosts within a single phage-host system suggesting that phages drive the diversification of bacterial populations within a given phylotype [41]. As the vast majority of prokaryotic taxa are not cultivable, culture-independent methods such as DGGE must be used to evaluate the effect of phage therapy on the total bacterial community structure.

The main goal of this work was to determine the survival of *A. salmonicida* and *V. parahaemolyticus* phages in marine water and to study their impact on the structure of the bacterial community of an aquaculture system of the Ria de Aveiro (Portugal).

## 2. Results and Discussion

### 2.1. Results

#### 2.1.1. Water Properties

In the aquaculture system, salinity varied between 31.0 PSU in March 2009 and 33.8 PSU in September 2009. Temperature varied from 13.8 °C in March 2009 to 20.2 °C in August 2009 and the dissolved oxygen ranged between 2.7 mg·L^−1^ in May 2009 and 1.7 mg·L^−1^ in July 2009.

#### 2.1.2. Phages Isolation and Classification

Two phages were isolated from samples of aquaculture water: *A. salmonicida* phages (AS-1) and *V. parahemolyticus* phages (VP-1).

**Figure 1:**
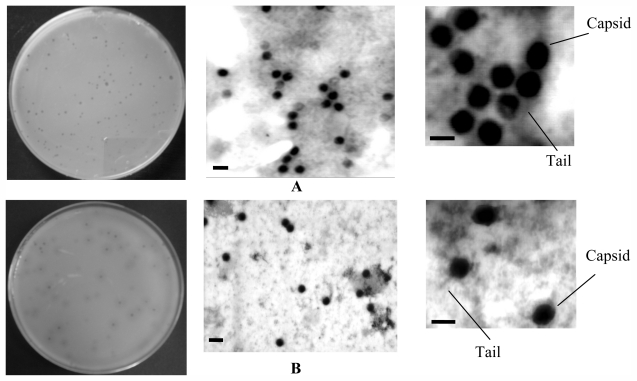
Phage plaques and electron micrographs of the phages. (**A**) VP-1 (scale bars = 50 nm); and (**B**) AS-1 (scale bars = 20 nm).

The presence of phages was conclusively confirmed by TEM examination of concentrated phage filtrates (Figure 1) [42]. Phage VP-1 and AS-1 appeared morphologically similar, exhibiting a tailed phage with an icosahedral head of approx. 50 and 40 nm diameter, respectively. Both isolated phages displayed binary symmetry and a long tail, approx. 100 nm in length. Phage isolates formed small plaques with a diameter varying between 1.5 mm and 2.0 mm after 6–8 h of incubation at 25 °C.

**Figure 2:**
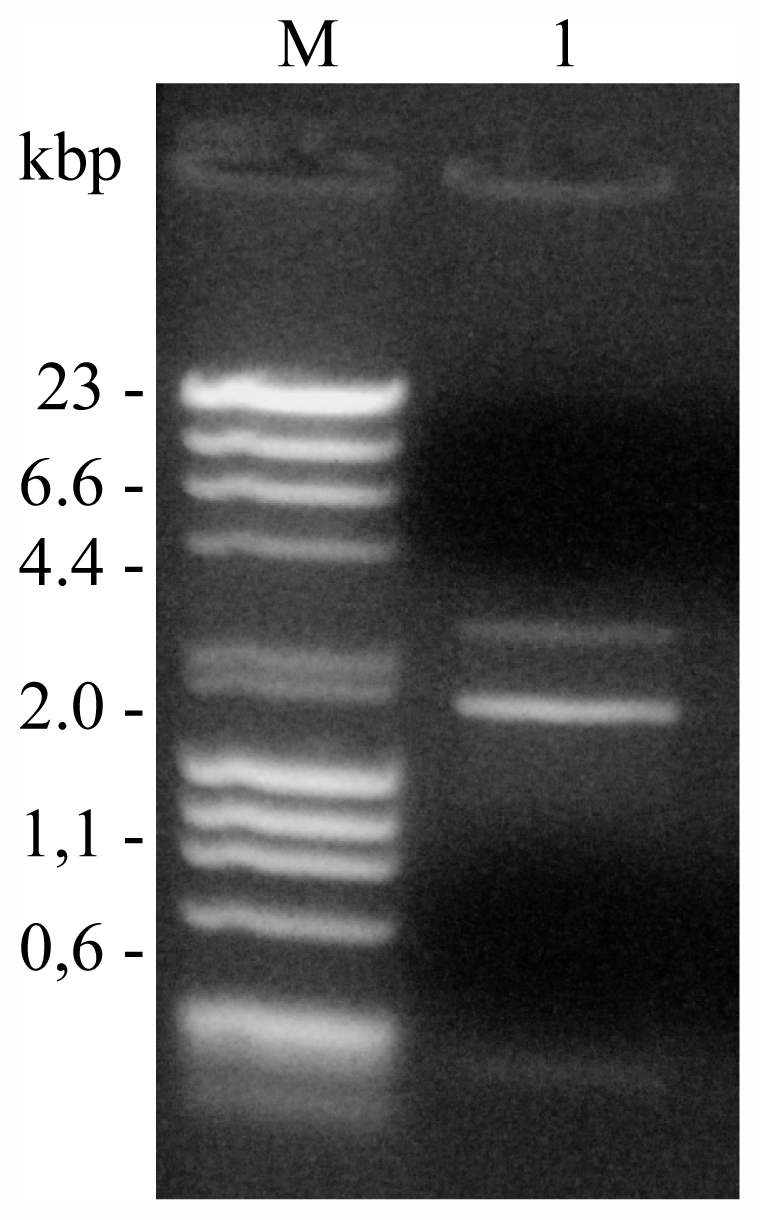
Phage VP-1 DNA following digestion with *Pst*I and *Bam*I. Lanes: M, Marker—DNA and DNA of bacteriophage fX174 digest with *Hin*d III and *Hae* III (Finnzymes); 1, VP-1 DNA digested with *Bam* I and *Pst*I.

The analysis of the nucleic acids of phages was conducted by three different methods: Strand Displacement Amplification (SDA) kit, enzyme digestion (DNase I, RNase I) and digestion with restriction enzymes (*Pst*I and *Bam*I). The nucleic acids of AS-1 and VP-1 were amplified by the SDA kit, as well as, digested by DNase I but not by RNase I, indicating that DNA is the nucleic acid. The nucleic acid of VP-1 was digested with *Pst*I and *Bam*I (Figure 2) in contrast with AS-1 that, after many attempts, was not cleaved by these enzymes. Nucleic acid analysis suggested that these phages have double stranded DNA genome.

#### 2.1.3. Phage Host Range Analysis

The phage VP-1 infected *Vibrio anguillarum* and *Aeromonas salmonicida* presenting an efficacy of 83.27 and 64.75, respectively (Table 1). The phage AS-1 infected *V. anguillarum* and *V. parahemolyticus* presenting an efficacy of 98.87 and 96.03%, respectively (Table 1). None of the nine phages was effective against *Photobacterium damselae* subsp. *damselae*, *Photobacterium damselae* subsp. *piscicida*, *Vibrio fischeri*, *Escherichia coli*, *Pseudomonas aeruginosa*, *Pseudomonas fluorescens*, *Pseudomonas putida*, *Pseudomonas gingeri* and *Pseudomonas segetis*.

#### 2.1.4. Determination of Phage Survival

**Figure 3:**
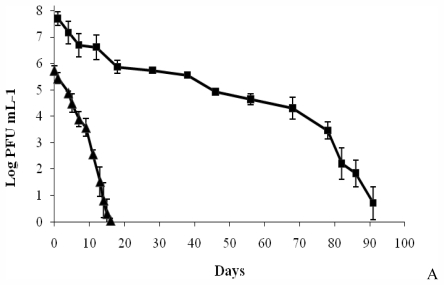
Survival of AS-1 and VP-1 phages. The values are expressed as the average of three independent experiments. Error bars represent the standard deviation. (■ AS-1 phage, ▴ VP-1 phage).

The pattern of phage survival in aquaculture water was different for the two phages tested. The abundance of AS-1 phage decreased by one order of magnitude in the first 15 days and, after reaching a plateau, that value remained constant during 45 days. Afterwards, the phage titer decreased slightly until 91 days (Figure 3). In contrast, the abundance of VP-1 phage decreased strongly during the incubation period, showing a survival period of 16 days, much lower than the AS-1 phage (Figure 3).

#### 2.1.5. Effects of the Phages on the Bacterial Community Structure

**Figure 4:**
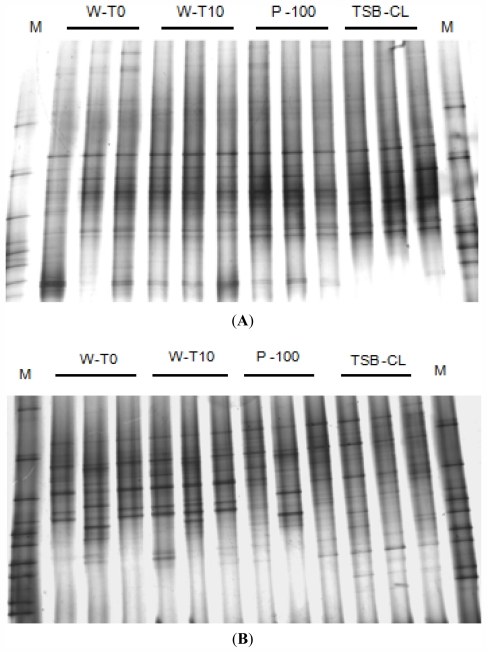
DGGE profile of PCR-amplified 16S rRNA gene fragments after AS-1 (**A**) and VP-1 (**B**) phages addition to bacterial community of the aquaculture system. M—molecular weight marker, W-T_0_—water samples at time zero; W-T_10_—water samples incubated during 10 h without phages; P-100—water samples added of 100 μL of AS-1 (**A**) and VP-1 (**B**) phages incubated during 10 h; TSB-CL—water samples incubated during 10 h with TSB and 1% of chloroform without phages.

**Figure 5:**
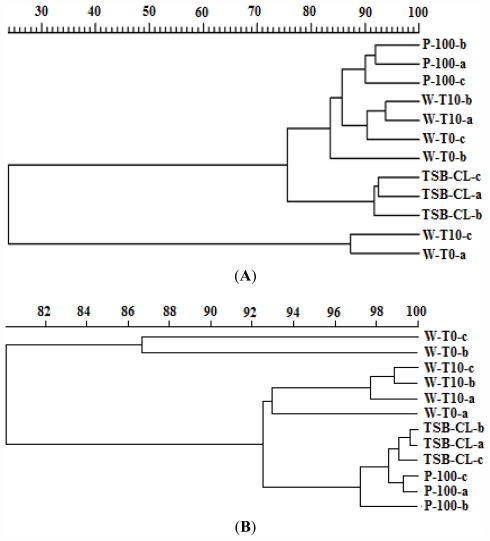
Dendrogram generated from the pattern of bands obtained by DGGE (Figure 3). (**A**) AS-1 and (**B**) VP-1. Cluster analysis was performed using the PRIMER v5 software [43]. The binary matrix was transformed into a similarity matrix using the Bray Curtis measure. W-T_0_—water samples at time zero; W-T_10_—water samples incubated during 10 h without phages; P-100—water samples added of 100 μL of AS-1 (**A**) and VP-1 (**B**) phages and incubated during 10 h; TSB-CL—water samples incubated during 10 h with TSB and 1% of chloroform without phages.

The effect of the addition of AS-1 and VP-1 phage on the structural diversity of the bacterial community of the aquaculture water as described by 16S rDNA DGGE profiles can be observed in Figures 4 and 5. The addition of the phages did not significantly alter the bacterial ribotype diversity. The pattern of bands in the sample with added phages (P-100) was similar to that of the control incubated during 10 h without phages (W-T_10_). The major difference was observed for the incubated controls with chloroform (TSB-CL) for *A. salmonicida* samples and for the non-incubated controls (W-T_0_) for *V. parahaemolyticus* samples (Figures 4 and 5).

Cluster analysis of the band patterns obtained from DGGE analysis of the experiment with AS-1 phage revealed the occurrence of two groups (Figure 4A). One that included water samples with added phages (P-100), non-added water samples incubated during 10 h (W-T_10_) and water samples at time zero (W-T_0_). The other includes water samples incubated during 10 h with TSB and chloroform without phages (TSB-CL). Samples W-T_0_, W-T_10_ and P-100 were closely grouped (similarity > 80%), but were separated from (TSB-CL) (similarity 75%).

Cluster analysis of the band patterns obtained from DGGE analysis of the experiment with VP-1 phage (Figure 4B) revealed the occurrence of two main groups. One including water samples with added phages (P-100), non-added water samples incubated during 10 h (W-T_10_) and water samples incubated during 10 h with TSB and chloroform (TSB-CL) with a similarity >92%. The other includes water samples at time zero (W-T_0_) with a similarity > 86%.

Analysis of similarities (ANOSIM) suggested moderated separation of bacterial communities treated with both tested phages (P100 *vs.* TSB-CL) (Table 2). However, the addition of TSB and 1% of chloroform without phages (TSB-Cl) to the microcosm samples and incubation time also resulted in significant separation of the communities.

### 2.2. Discussion

Fish infection by pathogenic bacteria is a progressive problem for the development of aquaculture worldwide. Several chemotherapies, such as the utilization of antibiotics, have contributed to a rapid and effective way to treat or prevent bacterial infections. However, the increasing problem of antibiotic resistance in common pathogenic bacteria and the concern about spreading antibiotics in the environment, bring the need of finding new methods to control fish pathogenic bacteria. Therefore, phage therapy represents a potentially viable alternative to antibiotics and to other antimicrobial compounds to inactivate indigenous and non-indigenous pathogenic bacteria in fish farming plants.

In this study, two phages were isolated from water aquaculture system, using isolated bacteria *V. parahaemolyticus* (phage VP-1) and *A. salmonicida* (phage AS-1) as hosts. TEM examination of the two presumptive phage isolates, indicated that they are assigned to the order Caudovirales because they display binary symmetry, long tail [42] and double stranded DNA [43].

The success of phage therapy in aquaculture depends mainly on the phages selected to inactivate the fish pathogenic bacteria [13]. The selected phages must remain viable in marine waters, infecting pathogenic bacteria but not altering significantly the non-pathogenic bacteria that have an important ecological role. The results of this study showed that both phages of fish pathogenic bacteria can survive in the aquaculture water at 25 °C temperature and that after 10 hour incubation they do not alter significantly the structure of the overall bacterial community. Unlike antibiotics, phages are self-replicating as well as self-limiting and, consequently, they replicate exponentially as bacteria replicate and decline when bacterial numbers decrease [27,44,45]. In this study it was possible to produce phages suspensions with high titers, up to 10^10^ PFU mL^−1^, suitable to use in phage therapy, that declined when bacteria were removed, but maintaining their high concentrations even after three months in marine water. These facts suggest that VP-1 and AS-1 phages are well adapted to the aquatic environment, surviving for long periods in marine waters and present a low ecological impact on the structure of the natural bacterial community. More extensive studies should be performed to test other incubation times and different phage concentrations on the aquaculture field.

Although it has been described that phages are specific to a single species or even strain of bacteria [46–48], the results of this study show that the VP-1 and AS-1 phages can inactivate pathogenic bacteria from different families with a high efficacy of plating. The phage VP-1 infected *V. anguillarum* and *A. salmonicida*, presenting an efficacy of 83.27 and 64.75%, respectively. The phage AS-1 infected *V. anguillarum* and *V. parahemolyticus* presenting a higher efficacy 95%. Phages of the indigenous bacterium *Vibrio*, which is responsible for the majority of the outbreaks in fish farms [6,12], infect their host family but also bacteria of other families, such as *Aeromonas*. Similar results were obtained by Miller *et al.* who isolated a broad-host-range vibriophage, KVP40, from sea water using *V. parahaemolyticus* (EB101), as the indicator host range of KVP40 extended over at least eight *Vibrio* sp. and one *Photobacterium* sp. [49]. Therefore, VP-1 and AS-1 phages can be tested for the ability to reduce the severity of different fish infections, namely *Vibrio* infections and furunculosis. However, these phages are not suitable to treat photobacteriosis. As the isolated bacteria, namely *V. parahaemolyticus*, display resistance to a broad range of antibiotics and are infected by the two viruses, phage therapy is a potentially viable alternative to antibiotics, inactivating even bacteria resistant to seven different antibiotics [50].

Although the phages VP-1 infect all the tested bacteria, they show only a moderate impact on the overall bacterial community structure. This fact contributes to the ecological balance of the microbial communities inhabiting the water of semi-intensive aquaculture regimes, where part of the fish food results from organic matter transformed and/or produced by aquatic bacteria. The number of ribotypes in water samples added of phages VP-1 after 10 h of incubation (P-100, Figure 4B) was similar to that obtained for water samples without phages addition after the same period of incubation (W-T_10_, Figure 4B). The pattern of bands obtained for water samples incubated during the 10 h (W-T_0_, P-100; TSB-CL) was, however, slightly distinct from those obtained for non-incubated samples (W-T_0_, Figure 4B) (Bray-Curtis similarity index of 80%). This difference can be explained by the bottle effect. It is well known that placing samples into containers terminates the exchange of cells, nutrients and metabolites with *in situ* surrounding environment [51], affecting bacterial community composition. The ANOSIM confirms these results, indicating only a moderate separation of bacterial communities after phage inoculation (P100 *vs.* TSB-CL, Table 2). The DGGE results for phages AS-1 show also a moderated effect on the structure of bacterial community. The bottle effect for these samples was not as evident as for the samples of *V. parahaemolyticus*. As the addition of both phages show only moderate impact on the dominant members of the total bacterial community structure, it will be interesting to test the effect of adding theses phages simply on specific bacterial groups, namely *Aeromonas* and *Vibrio*, using primers specific for these groups in the DGGE approach instead of the universal primers used in this study.

Previous studies in this aquaculture system [52] showed that the bacterial community structure of total and pathogenic bacterial communities varied seasonally, showing a higher complexity during the warm season. The authors concluded that: (1) the seasonal variation of the bacterial communities imply the need for a careful monitoring of water throughout the year in order to select suitable phages to inactivate fish pathogenic bacteria; and (2) that the spring season seems to be the critical time period when phage therapy should be applied. Consequently, the impact of the phages on the structure of the bacterial community can also vary seasonally. However, the study of the impact of the phages on the bacterial community was conducted during the warmer seasons which is the critical time period when phage therapy should be applied [52].

Further studies should be performed to select the most effective phage strain or effective combination of phage strains for therapeutic applications. It will be also important to characterize the capacity of phages to reduce their host fitness. Moreover, it should be emphasized that before using bacteriophages for therapy, it would be important to test whether they carry any virulence genes, that is, if there is any potential for lysogenic conversion.

## 3. Experimental Section

### 3.1. Study Area and Sampling

This study was conducted in the semi-intensive aquaculture system Corte das Freiras located in the estuarine system Ria de Aveiro (latitude: 40°37′51.44′N, longitude 8°40′31.75′ W) on the north-western coast of Portugal. Since the aquaculture is located near the city of Aveiro it is subjected to some contamination introduced by human wastes and, therefore subjected to chemotherapy treatment. The aquaculture is divided in ten earthen ponds of approximately 2500 m^2^ each, which are supplied with water from Ria de Aveiro. The *Sparus aurata* (gilthead seabream) and *Dicentrarchus labrax* (European seabass) cultured in this aquaculture system are stocked at 12,000 fish/ha.

Water samples were collected between March 2009 and September 2009, two hours before low tide, in mild weather conditions, from a culture tank of *Sparus aurata*. Samples from surface water were taken directly into sterile glass bottles and kept cold and in the shade during transport to the laboratory where they were processed within the next 1–2 h.

### 3.2. Water Properties

Temperature and salinity were measured in the field using a WTW LF 196 Conductivity Meter. Dissolved oxygen was also determined in the field with a WTW OXI 96 oxygen meter equipped with a WTW BR 190 stirrer. pH was measured in the laboratory, at 25 °C, with a pH probe (Orion, Model 290 A).

### 3.3. Microorganisms and Growth Conditions

The three bacterial strains *V. anguillarum*, *V. parahaemolyticus* and *A. salmonicida* used in this study were previously isolated in our laboratory from the aquaculture systems Corte das Freiras of Ria de Aveiro (Portugal) [50]. The other nine strains, *P. damselae* subsp*. damselae* (ATCC 33539), *P. damselae* subsp. *piscicida* (ATCC 29690) [18], *E. coli* [53], *V. fischerie* (ATCC 49387), *P. aeruginosa*, *P. fluorescens*, *P. putida*, *P. segetis* and *P. gingeri* [54], used in this work were obtained in previous studies. The bacteria were stored at 4 °C in tryptic soy broth (TSB, Merck). Before each assay the strains were grown aerobically for 24 h at 25 °C in 30 mL of TSB. Then, aliquots of these cultures (300 μL) were aseptically subcultured to 30 mL of fresh TSB medium and grew overnight at 25 °C.

### 3.4. Bacteriophages Isolation and Purification

Bacteriophages were isolated from aquaculture water, collected at Corte Freiras (Ria Aveiro, Portugal). Two pathogenic bacteria of fish (*V. parahaemolyticus* and *A. salmonicida*) were used as hosts to produce the phage suspensions.

Five hundred milliliters of water was filtered sequentially by 3 μm and then 0.22 μm-pore-size polycarbonate membranes (Poretics). The filtered water was added to five hundred milliliters of TSB with double concentration and one milliliter of the respective bacterial host suspension in exponential growth. Suspensions were incubated overnight at 25 °C, with shaking (100 rpm min^−1^) and were then centrifuged at 9000 g for 10 min (rotor JA.25.50, Beckman Avanti^TM^ J-25I). The supernatant was then filtered through 0.22 μm pore-size polycarbonate membranes. The spot test method, a procedure based on the double layer plaque technique [55] with minor modifications, was used as an initial test for the presence of phages. Three milliliters of TSB 0.6% agar (Merck), previously inoculated with 300 μL of a specific bacterial culture with 8–10 h were overlaid on solid tryptone soy agar (TSA, Merck) and spotted with 10 μL of the filtered suspension. Petri plates were incubated at 25 °C for 6–8 h. A clear zone in the plate, resulting from the lysis of host bacterial cells, indicated the presence of phages.

In order to isolate phages from this clear lysis zone, serial dilutions in phosphate buffered saline (137 mmol^−l^ NaCl (Sigma), 2.7 mmol L KCl (Sigma), 8.1 mmol^−1^ Na_2_HPO_4_·2H_2_O, 1.76 mmol^−1^ KH_2_PO_4_ (Sigma), pH 7.4) were prepared from the phage stocks obtained above. A colony of the respective host strain was grown 3–4 h (early-log phase culture) in 5 mL of TSB. A volume of 500 μL of phage-containing sample and 100 μL of host culture were mixed with 3 mL of 0.6% TSB agar, overlaid onto TSA plates and incubated at 25 °C for 6–8 h. Phages were purified by successive single plaque isolation, from the higher dilutions plates where plaques were still distinct. A single plaque was picked from the bacteria lawn, inoculated into an early-log phase host culture, and the lysate plated as described above. After repeating the cycle two more times, lysates from single plaques were treated with 1% chloroform, mixed and centrifuged at 9000 g for 5 min. The phages were recovered from the upper phase suspension and filtered through 0.22 μm membranes. Phages stocks were stored at 4 °C.

The number of phages present in this suspension was determined using the soft agar overlay technique, according to Adams [56]. Successive dilutions of the phage suspension were performed in a phosphate buffered saline and 500 μL of each dilution together with 100 μL of the respective bacterial host culture were mixed with 3 mL of TSB 0.6% top agar layer and placed over a TSA plate. The plates were incubated at ambient temperature (25 °C) for 6–8 h, the number of plaques was counted and the results expressed as plaque forming units per milliliter (PFU mL^−1^). Phage titration was performed in triplicate.

### 3.5. Sample Preparation for Transmission Electron Microscopy (TEM)

Each phage suspension (10 mL) was centrifuged directly onto formvar-coated carbon-stabilized 400 mesh copper electron microscopy grids as previously described [57]. The viruses were ultracentrifuged at 100,000 g for 1 h 30 min at 25 °C in a Beckman L8-80K ultracentrifuge equipped with a swing-out rotor (SW28). After centrifugation, the grids were rinsed briefly with distillated water, negatively stained with 1.5% (w/v) uranyl acetate for 60 seconds [58] air-dried and examined with a JEOL 100CX transmission electron microscope at a magnification of 100,000 × and an accelerating voltage of 100 kV.

### 3.6. Concentration of Bacteriophage Particles with PEG for Nucleic Acid Extraction

Precipitation of bacteriophage particles with polyethylene glycol (PEG) was used as described by Sambrook and Russel [59]. PEG 8000 was added to the centrifuged lysate and gently mixed to dissolve. The lysates were incubated at 4 °C for 60 min and the precipitated particles pelleted by spinning at 11,000 g for 10 min. Pellets were resuspended in TE buffer [10 mM Tris HCl, 1 mM ethylenediamine tetraacetic acid (EDTA), pH 8.0]. The phage and bacteria debris was extracted with chloroform and the aqueous phase was collected.

### 3.7. Nucleic Acid Extraction and Amplification of Phage DNA Using Phi29 DNA Polymerase

The extraction of nucleic acid from phage particles was conducted as described by Griffiths *et al.* [60]. Extractions were performed by the addition of 0.5 mL of hexadecyltrimethylammonium bromide (CTAB) extraction buffer and 0.5 mL of 0.5 mL of phenol-chloroform-isoamyl alcohol (25:24:1) (pH 8.0) CTAB. Samples were lysed for 30 s in a FastPrep FP120 (BIO 101/Savant) at a speed of 5.5 ms^−1^, and the aqueous phase containing nucleic acids was separated by centrifugation (16,000 g) for 5 min at 4 °C. The aqueous phase was then extracted and phenol was removed by mixing with an equal volume of chloroform-isoamyl alcohol followed by repeated centrifugation (16,000 g) for 5 min at 4 °C. Total nucleic acids were subsequently precipitated from the extracted aqueous layer with 2 volumes of 30% (wt/v) polyethelene glycol–1.6 M NaCl for 2 h at room temperature, followed by centrifugation (18,000 g) at 4 °C for 10 min. Pelleted nucleic acids were then washed in ice cold 70% (v/v) ethanol and air dried prior to resuspension in 50 mL of TE buffer (10 mM Tris HCl, 1 mM ethylenediamine tetraacetic acid (EDTA), pH 8.0).

Nucleic acid yield was estimated using NanoDrop ND-1000 spectrophotometer (version 3.3). DNA purification was performed using the Geneclean spin kit (MP Biomedicals, LLC).

The TempliPhi DNA Sequencing Template Amplification kit (Amersham Biosciences) was used as described in the manufacturer protocol booklet with the following exceptions. The template (2 μL) was added to a mixture of 6 μL of sample buffer and 2 μL of dd-water. During the denaturation step, a reaction premix of 12 μL reaction buffer + 0.8 μL BSA (0.25 μg/μL) + 0.5 μL enzyme mix, for each sample was prepared. Once tubes were cooled from denaturation temperature, 7 μL of this freshly made premix was added to each sample and were incubated at 30 °C for 4.5 h. The reaction was terminated at 65 °C for 10 min. Samples were analyzed using 0.8% agarose gel electrophoresis at 80 V.

### 3.8. Nucleic Acid Characterization

The nucleic acid extracts were digested with DNase I (Ambion), RNase I (Sigma Aldrich), *Bam*I (Fermentas) and *Pst*I (Fermentas) as described by the manufacturer. The reactions were terminated by heating at 80 °C for 5 min. Nucleic acid yield was observed through agarose gel electrophoresis (0.8% agarose gel electrophoresis at 80 V).

### 3.9. Phage Host Range Analysis

Bacterial susceptibility to bacteriophage was assayed for the five pathogenic bacteria of fish (*V. parahaemolyticus*, *A. salmonicida*, *V. anguillarum*, *P. damselae* subsp. *damselae* and *P. damselae* subsp. *piscicida*). The spot test method was used as an initial approach for the detection of bacterial infection [55]. The efficiency of plating was determined for the bacteria with positive spot tests (occurrence of lysis plaques) using the soft agar overlay technique [56]. Efficacy of plating for each host was calculated by comparison with an efficacy of 100% for the *V. parahaemolyticus* and *A. salmonicida* phages on the *A. salmonicida* and *V. parahaemolyticus* bacteria, respectively. For each phage three independent experiments were done and the results presented are the average of the three assays.

### 3.10. Determination of Phage Survival

The survival of VP-1 and AS-1 phages was tested in aquaculture marine water collected on July and August 2009. One hundred milliliters of water were filtered through 3 μm and then by 0.22 μm pore-size membranes (Poretics) and 50 μL of phage suspension was added, followed by incubation at 25 °C with shaking (100 rpm·min^−1^) in the dark. Phage titer was determined at time zero and at intervals of 72 h, using the soft agar overlay technique [56]. The plates were incubated at ambient temperature (25 °C) for 6–8 h. For each phage three independent experiments were done and the results presented are the average of the three assays. The results are presented by survival curves plotted as logarithmic phages reduction (log PFU·mL^−1^) *vs.* time incubation (in days).

### 3.11. Impact of Phage Addition on Bacterial Community Structure

For each phage, 150 mL of aquaculture water, collected on June and July 2009, were added to each of 12 erlenmeyers. Each of the phages was added to three erlenmeyer flasks at a final concentration of 4.1 × 10^6^ PFU·mL^−1^ for the AS-1 phage and 1.1 × 10^7^ PFU·mL^−1^ for the VP-1 phage P-100. Three negative controls were also included (n = 3 flasks each) to control for the phage preservative solution (TSB with 1% chloroform; TSB-CL), a water incubation without added phage (W-T_10_), and water control without incubation (W-T_0_). The P-100, TSB-CL and W-T_10_ were incubated at 25 °C during 10 h. After incubation, each sample was filtered through 0.22 μm pore-size filters (Poretics). W-T_0_ samples were not incubated, being immediately filtered.

For the extraction of bacterial DNA, the bacterial cells retained on the membranes were resuspended in 2 mL of TE buffer (10 mM Tris HCl, 1 mM ethylenediamine tetraacetic acid (EDTA), pH 8.0) and centrifuged. After resuspension in 200 mL TE, 2 mg mL^−1^ lysozyme solution was added to induce cell lysis and incubated at 37 °C for 1 h, according to the procedure described by Henriques *et al.* [61]. DNA was resuspended in TE buffer and stored at −20 °C.

The DNA extracted was used to amplify 16 rRNA gene fragments, using a nested PCR approach. In the first PCR, the universal bacterial primers 27F and 1494R were used to amplify *ca.* 1450 bp of the 16S rRNA gene [62]. The reaction was carried in a Multigene Gradient Thermal Cycler from MIDSCI. A reaction mixture of 25 μL was prepared containing 1 × PCR buffer (Fermentas), 0.2 mM deoxynucleoside triphosphates, 3.75 mM MgCl_2_, 4% (v/v) bovine serum albumin (BSA, Sigma), 0.1 μM primers synthesized by IBA, 1 U Taq polymerase (Fermentas), and template DNA (*ca*. 10 ng). After 5 min of denaturation at 94 °C, 30 thermal cycles of 45 s at 94 °C, 45 s at 56 °C, and 1.5 min at 72 °C were carried out. A final extension step at 72 °C for 10 min was performed to finish the reaction. One microlitres of the product of the first PCR was used as the template for a second PCR with bacterial DGGE primers F968-GC (5′-GC-clamp-AACGCGAAGAACCTTAC-3′) and R1401 (5′-GCGTGTGTACAAGACCC-3′) [63], according to the procedure described by Pereira *et al.* [52]. PCR products were checked using standard agarose gel electrophoresis (0.8% agarose, 1× TAE buffer; 100 V for 40 min) and ethidium bromide staining [64].

Samples containing approximately equal amounts of PCR amplicons were analyzed by DGGE, performed with a CBS System (CBS Scientific Company, Del Mar, CA, USA). PCR products were loaded onto 6–9% polyacrylamide gel in 1xTAE buffer (20 mmol·L^−1^ Tris, 10 nmol·L^−1^ acetate, 0.5 mmol·L^−1^ EDTA pH 7.4). The 6–9% polyacrylamide gel (bisacrylamide:acrylamide = 37.5:1) was made with a denaturing gradient ranging from 32 to 60%. Electrophoresis was performed at 60 °C for 16 h at 150 V. Following electrophoresis, the gels were silver stained, according to the procedure described by Pereira *et al.* [52].

The gels were digitalized and analyzed with the software package Gelcompar 4.0 program (Applied Maths) as previously described by Smalla *et al.* [65]. After automatic band search, the bands detected were carefully checked and artefacts were removed. The sets used for band detection were 5% minimal profiling (area along the densitometric curve) and 0.5% minimal area. The positioning and quantification of bands were carried out by setting tolerance and optimization at 5 points, *i.e.*, 1.0%. The band positions and their corresponding intensities from each treatment were exported to Excel files and the band surface was converted to relative intensity by dividing its surface by the sum of all band surfaces in a lane. Bray-Curtis similarities were calculated based on the band position and intensity. The matrices of similarities were then used for multivariate analyses of DGGE profiles using analysis of similarities (ANOSIM) with the PRIMER 5 software package (Primer-E Ltd., Plymouth, UK). The *R* value in ANOSIM ranges from 0 to 1, where *R* > 0.75 indicates significant differences, *R* > 0.5 moderate separation and *R* < 0.25 high similarity [42].

## 4. Conclusions

The results suggest that the bacterial phages tested in this study have long term survival in marine water and show only a moderate impact on the overall bacterial community structure. Moreover, the wide host range of VP-1 and AS-1 improves their potential to inactivate a broader range of fish pathogenic bacteria. Therefore, the phages investigated in this study are potential candidates for development of phage therapy directed to the prophylaxis of disease outbreak and/or treatment of symptomatic diseases in marine aquaculture systems. Further studies should be performed to select the most effective phage strain or effective combination of phage strains for therapeutic applications. Experiments to determine the effectiveness of phage against natural infections should also be performed in order to develop an efficient protocol to treat fish bacterial infections.

## Figures and Tables

**Table 1:** Efficacy of plating (%) to different fish pathogenic bacteria.

FISH PATHOGENIC BACTERIA	PHAGES	

	AS-1	VP-1
*V. anguillarium*	98.87	83.27
*V. parahaemolyticus*	96.03	100
*V. fischeri*	0	0
*A. salmonicida*	100	64.75
*P. damselae* subsp. *damselae*	0	0
*P. damselae* subsp. *piscicida*	0	0
*E. coli*	0	0
*P. aeruginosa*	0	0
*P. fluorescens*	0	0
*P. putida*	0	0
*P. segetis*	0	0
*P. gingeri*	0	0

**Table 2:** ANOSIM pairwise comparison of DGGE fingerprints of 16S rRNA gene fragments after AS-1 (**A**) and VP-1 (**B**) phages addition to the microcosms. W-T_0_—water samples at time zero; W-T_10_—water samples incubated during 10 h without phages; P-100—water samples added of 100 μL of AS-1 and VP-1 phages and incubated during 10 h; TSB-CL—water samples incubated during 10 h with TSB and 1% of chloroform without phages.

Groups	*R*	

	AS-1	VP-1
WT_0_, WT_10_	0.037	0.333
WT_0_, TSB-CL	0.667	0.778
WT_0_, P_100_	0.333	0.556
TSB-CL, P_100_	0.630	0.519
TSB-CL, WT_10_	0.963	0.407
P_100_, WT_10_	0.556	0.185
